# Ultrasound-Guided Selective Trunk Block Combined With Supraclavicular Nerve Block for Awake Shoulder Arthroscopy: A Case Series

**DOI:** 10.7759/cureus.107996

**Published:** 2026-04-29

**Authors:** Neha Rani, Krishan Kumar, Neha Shrivastava, Jaiveer Singh, Vineet Purthi

**Affiliations:** 1 Anesthesiology, Employees' State Insurance Corporation (ESIC) Medical College and Hospital, Faridabad, IND; 2 Orthopedics Sports Medicine, Employees' State Insurance Corporation (ESIC) Medical College and Hospital, Faridabad, IND; 3 Orthopedics, Employees' State Insurance Corporation (ESIC) Medical College and Hospital, Faridabad, IND

**Keywords:** regional blocks, rotator cuff tears, sports surgery, supraclavicular block, ultrasonography

## Abstract

The evolution of shoulder arthroscopy from inpatient to outpatient settings has necessitated the development of advanced regional anesthesia techniques that provide reliable intraoperative analgesia and hemodynamic stability while minimizing respiratory complications associated with conventional interscalene brachial plexus blocks, which are often linked to unintended phrenic nerve blockade and resultant diaphragmatic dysfunction. This prospective case series (level of evidence IV) aimed to evaluate the surgical efficacy, patient tolerance, and preservation of phrenic nerve function using a hybrid regional anesthesia approach combining ultrasound-guided selective trunk block (SeTB) and supraclavicular nerve (SCN) block. Twenty-eight ASA I-II patients undergoing elective arthroscopic shoulder surgery received SeTB combined with SCN block using 23 mL of 0.5% bupivacaine. The technique achieved a 100% surgical success rate, with a mean operative duration of 1.88 ± 0.58 hours and a mean reduction in diaphragmatic excursion of 20.8 ± 1.7%. Overall, SeTB-SCN provided effective phrenic-sparing anesthesia for awake shoulder arthroscopy.

## Introduction

The field of orthopedic sports medicine has undergone a profound transformation in recent decades, driven by advancements in minimally invasive techniques and a growing emphasis on patient-centered outcomes. Shoulder arthroscopy, a cornerstone procedure for addressing common athletic injuries such as rotator cuff tears, glenoid labral instability, and biceps tendon pathologies, exemplifies this shift. Historically performed under general anesthesia in hospital settings, these surgeries are increasingly being performed in ambulatory, outpatient settings to reduce healthcare costs, minimize hospital-acquired complications, and expedite functional recovery [1-3]. This evolution is particularly relevant for sports medicine practitioners, who care for active individuals and athletes who require a swift return to training and competition.

Central to this transition is the optimization of anesthetic strategies. General anesthesia, while reliable, carries inherent risks, including postoperative nausea, cognitive fog, prolonged recovery times, and potential airway complications, issues that are especially problematic for athletes prioritizing rapid rehabilitation [1,4]. Regional anesthesia techniques, particularly brachial plexus blocks, have emerged as preferred alternatives for enabling "awake" surgery, in which patients remain conscious and cooperative, thereby facilitating immediate postoperative assessment and discharge [2,5].

However, the standard interscalene block (ISB), which targets the brachial plexus at the C5-C6 root level, is associated with a near-ubiquitous risk of hemidiaphragmatic paralysis (HDP). This occurs because the phrenic nerve, originating from C3-C5 roots, lies in close anatomical proximity to the injection site, leading to unintended blockade in up to 100% of cases [6]. HDP manifests as a significant reduction in diaphragmatic excursion (DE), often exceeding 75%, which can cause dyspnea, hypoxemia, and contraindicate outpatient procedures in patients with pre-existing respiratory conditions or those needing preserved ventilatory capacity for early mobilization [7]. For high-performance athletes, even transient respiratory impairment can delay return to sport, underscoring the need for phrenic-sparing innovations.

The selective trunk block (SeTB), described by Karmakar et al., is a novel ultrasound-guided technique that individually targets the superior, middle, and inferior trunks of the brachial plexus to provide upper limb anesthesia while aiming to preserve diaphragmatic function [5]. Hong et al. further refined this approach by using a single skin entry with transducer repositioning to sequentially block each trunk, thereby improving patient comfort, particularly in awake procedures [4]. SeTB is a distal approach targeting the brachial plexus at the trunk level, approximately 2-3 cm caudal to the roots, where the plexus diverges from the anterior scalene muscle and the phrenic nerve [8]. This anatomical shift theoretically reduces phrenic involvement by allowing precise local anesthetic deposition away from sensitive structures. Early reports suggest that SeTB provides adequate analgesia for upper-extremity procedures while preserving diaphragmatic function [9].

Subsequent investigations into the minimum effective volume required for SeTB further support its feasibility and safety in clinical practice. However, SeTB alone may not adequately cover the “cape” area (C3-C4 dermatomes), which is innervated by the supraclavicular nerve (SCN). The addition of an SCN block enhances analgesic coverage for shoulder procedures, particularly in awake arthroscopy. Without this adjunct, patients may experience residual pain in the cape region, potentially disrupting surgical flow and necessitating supplemental sedation or opioids [10-12]. Furthermore, in the lateral position, forearm traction can induce muscle spasm and discomfort [1]. The comprehensive blockade of the shoulder and upper limb achieved with the combined SeTB and SCN block effectively mitigates these effects.

To overcome this limitation, we introduce a hybrid technique combining SeTB with a targeted SCN block. Building on prior clinical experience with SeTB in awake procedures [13], we conducted a prospective case series of patients undergoing arthroscopic shoulder surgery in the lateral position, using a low-volume, ultrasound-guided SeTB (modified per Hong et al.) with an SCN block. The primary objective was to determine whether this combined SeTB-SCN technique provides effective surgical anesthesia for awake shoulder arthroscopy while preserving diaphragmatic function. Secondary objectives included assessing patient tolerance during awake surgery, evaluating the duration and quality of postoperative analgesia, determining perioperative opioid requirements, and measuring (DE) as a surrogate marker of phrenic nerve involvement using M-mode ultrasonography.

## Materials and methods

Study design and ethical considerations

This prospective observational case series was conducted at ESIC Medical College and Hospital, Faridabad, Haryana, India, a tertiary-care academic medical center. Ethical approval was obtained from the Institutional Ethics Committee (approval number: 134 X/11/13/2023-IEC/DHR/47).

Patient selection and preoperative evaluation

Participants were recruited consecutively from the orthopedic outpatient clinic between February 2025 and December 2025. Inclusion criteria encompassed adults aged 18-65 years with ASA physical status I or II, scheduled for elective unilateral arthroscopic shoulder surgery under regional anesthesia. Specific procedures included rotator cuff repair (n = 12), glenoid labral stabilization (n = 10), and biceps tenodesis (n = 6), all of which are common in sports-related injuries. Exclusion criteria were meticulously defined to ensure patient safety: pre-existing phrenic nerve or diaphragmatic dysfunction (e.g., from prior trauma or neuromuscular disease), morbid obesity (body mass index (BMI) >35 kg/m² due to increased risk of respiratory complications and ultrasound visualization challenges), known allergy to local anesthetics, coagulopathy, infection at the injection site, or refusal to provide informed consent. Additionally, patients with severe cardiopulmonary comorbidities (e.g., chronic obstructive pulmonary disease with FEV1 <50% predicted) were excluded to avoid confounding respiratory outcomes.

All eligible patients underwent a detailed preoperative assessment, including a medical history review, a physical examination, and baseline pulmonary function tests, if indicated. Written informed consent was obtained after explaining the procedure, potential risks (e.g., nerve injury <1% and local anesthetic systemic toxicity (LAST) <0.2%), benefits, and alternatives. Patients adhered to ASA fasting guidelines (nil per os for six hours for solids and two hours for clear liquids) to minimize the risk of aspiration, even though general anesthesia was not planned.

Baseline diaphragmatic function was quantitatively evaluated using M-mode ultrasonography (SonoSite M-Turbo, SonoSite, Inc., USA), a non-invasive and reproducible method for assessing DE [7,12]. A 3.5-5 MHz convex probe was positioned subcostally along the anterior axillary line via hepatic (right side) or splenic (left side) windows during quiet and deep breathing. DE was measured as the vertical distance from baseline to peak excursion during maximal inspiration, averaged over three cycles to account for variability. This preoperative metric served as the reference for postoperative comparisons.

Anesthetic technique and surgical positioning

All blocks were performed in a dedicated preoperative block room by two senior anesthesiologists with over 05 years of experience in ultrasound-guided regional anesthesia, ensuring consistency and minimizing operator-dependent variability. Patients were monitored with standard ASA equipment (electrocardiography, non-invasive blood pressure, and pulse oximetry) and received supplemental oxygen (2 L/min via nasal cannula) if SpO₂ dropped below 95%. Mild sedation with midazolam (1-2 mg IV) was administered if requested for anxiety relief, but was avoided routinely to maintain the "awake" state.

The hybrid block employed a modified single-skin entry technique to enhance procedural efficiency and patient comfort [3,8]. Using aseptic technique and skin infiltration with 2% lidocaine (2 mL), a SeTB combined with SCN block was performed with a high-frequency ultrasound system and a 6-15 MHz linear transducer, using a 21G, 100 mm insulated Stimuplex A needle (B. Braun, Melsungen, Germany). The patient was positioned supine with the head turned contralaterally. Under continuous in-plane ultrasound guidance, the probe was initially placed in the supraclavicular fossa in a transverse orientation to identify the subclavian artery, first rib, and brachial plexus elements (trunks/divisions). The inferior trunk (IT) was targeted at the “corner pocket” (junction of the subclavian artery and first rib). Needle position was confirmed by hydrodissection, and 10 mL of 0.5% bupivacaine was injected in 2-3 mL increments with intermittent aspiration. Without withdrawing the needle from the skin, the medial end of the probe was rotated cephalad to visualize the superior trunk (ST) and middle trunk (MT) within the interscalene groove, maintaining an oblique transverse orientation. The needle was redirected in-plane, and 5 mL of 0.5% bupivacaine was deposited around each trunk in 1-2 mL increments with hydrodissection to ensure adequate spread and avoid intraneural injection [9,10].

**Figure 1:**
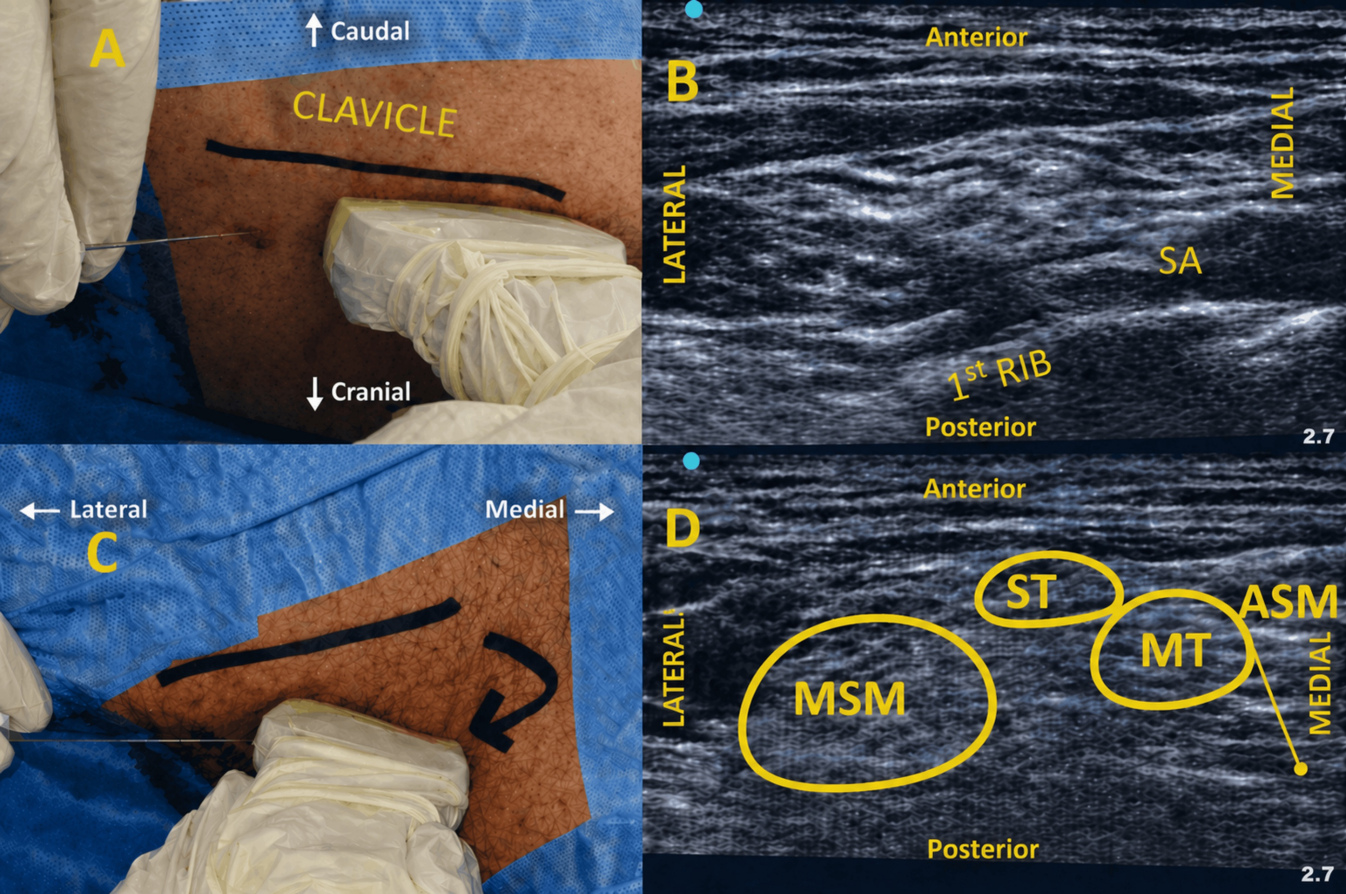
Ultrasound-guided SeTB technique (A) Patient positioned supine with the head turned contralateral to the block side. The ultrasound transducer is placed in the supraclavicular fossa to identify the inferior trunk in the corner pocket. (B) Corresponding sonographic image demonstrating the inferior trunk (IT) adjacent to the SA over the first rib. (C) Cephalad rotation of the medial end of the ultrasound transducer, without changing the lateral end or needle entry point, allows visualization of the ST and MT in the interscalene groove. (D) Corresponding sonographic image showing the ST and MT located between the ASM and the MSM. SA: subclavian artery, ASM: anterior scalene muscle, MSM: middle scalene muscle, ST: superior trunk, MT: middle trunk, IT: inferior trunk, SeTB: selective trunk block

Following truncal injections, the needle was withdrawn and repositioned in-plane at the midpoint of the posterior border of the sternocleidomastoid muscle. The SCN block was performed by depositing 3 mL of 0.5% bupivacaine in the fascial plane between the sternocleidomastoid (deep investing fascia) and middle scalene muscle (prevertebral fascia), administered in 0.5 mL aliquots [10]. Total local anesthetic volume was capped at 23 mL to minimize the risk of LAST, with aspiration checks and incremental injection (Figure 1). Block efficacy was assessed serially every five minutes using a validated 0-2 grade scale for sensory (pinprick) and motor (shoulder abduction/adduction) function in relevant dermatomes/myotomes (C5-T1) [11,13].

**Figure 2:**
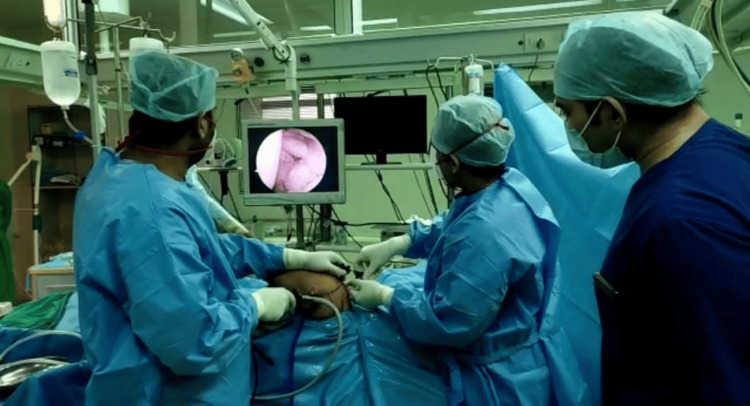
Awake shoulder arthroscopy in lateral decubitus position Intraoperative photograph demonstrating shoulder arthroscopy performed in the lateral decubitus position with forearm traction under regional anesthesia.

Grade 2 (complete loss) was required for proceeding to surgery. Upon confirmation, patients were transferred to the operating room and positioned in lateral decubitus with the operative shoulder uppermost, using a beanbag for stability and 10-15 lbs of balanced forearm traction via a specialized arm holder to optimize glenohumeral joint exposure (Figure 2).

Intraoperative management and postoperative protocol

Intraoperative maintenance focused on patient comfort and hemodynamic stability. Intravenous paracetamol (1 g) was administered for multimodal analgesia, supplemented by a low-dose dexmedetomidine infusion (0.2-0.7 μg/kg/h) for anxiolysis without respiratory depression (Ramsay sedation score 2-3) [14]. Breakthrough pain was managed with fentanyl boluses (25-50 μg IV), titrated to effect, while avoiding deep sedation. Surgical procedures were performed by experienced orthopedic surgeons using standard arthroscopic techniques: anterior, posterior, and lateral portals for visualization, debridement, suture anchor placement, and tissue repair as indicated.

Postoperatively, patients were transferred to the post-anesthesia care unit for continuous monitoring. DE was reassessed 30 minutes later by the same anesthesiologist, using the same M-mode ultrasound protocol, to minimize interobserver variability. HDP was defined as a ≥25% reduction from baseline, a threshold correlated with clinical symptoms [7,12]. Patients were monitored in the post-anesthesia care unit for two hours, assessing for complications such as Horner syndrome, hoarseness, or LAST signs (e.g., tinnitus, seizures). Pain was quantified using the VAS (0-10 scale) at 6, 12, and 24 hours postoperatively [15]. Rescue analgesia (oral tramadol 50 mg) was provided for VAS >4. Discharge criteria included stable vital signs, VAS <3, and independent ambulation, with the goal of same-day outpatient release.

Statistical analysis

Data were analyzed descriptively using SPSS Statistics version 25.0 (IBM Corp. Released 2017. IBM SPSS Statistics for Windows, Version 25.0. Armonk, NY: IBM Corp.). Continuous variables (e.g., DE reduction, VAS scores) are presented as mean ± standard deviation (SD), while categorical outcomes (e.g., surgical success) are presented as frequencies and percentages. No inferential statistics were applied, given the case series design, but trends were noted to inform hypothesis generation in future studies.

## Results

Baseline patient characteristics are summarized in the Appendices. The study cohort comprised 28 patients with a mean age of 44.8 ± 17.5 years (range 22-62 years), including 18 males and 10 females. BMI averaged 26.4 ± 4.2 kg/m². All patients achieved complete grade 2 sensory-motor blockade within 15.9 ± 3.0 minutes post-injection, enabling prompt commencement of surgery. The mean surgical duration was 1.88 ± 0.58 hours (range 1.2-2.8 hours), with no intraoperative complications such as vascular puncture or paresthesia reported.

Surgical success, defined by no conversion to general anesthesia or need for any rescue block, was 100%, highlighting the technique's reliability across procedure types. Intraoperative stability was excellent; hemodynamic parameters remained within 20% of baseline, and breakthrough pain was minimal, requiring only 56.1 ± 12.4 μg of fentanyl per case, far below typical opioid needs in comparable surgeries under ISB or general anesthesia.

Respiratory outcomes were particularly favorable. Preoperative DE averaged 5.2 ± 0.8 cm during deep inspiration. Postoperative assessment revealed a mean DE reduction of 20.8 ± 1.7% (range 18-23%), well below the 25% HDP threshold, with no patients experiencing dyspnea or requiring supplemental oxygen beyond routine. This preservation of diaphragmatic function underscores the phrenic-sparing advantage.

Postoperative analgesia was robust, with time to first rescue analgesic averaging 17.0 ± 2.9 hours. VAS scores demonstrated effective pain control: 0.2 ± 0.6 at six hours, 1.2 ± 1.7 at 12 hours, and 4.0 ± 1.4 at 24 hours, allowing all patients to be discharged on the same day without readmissions. No adverse events, including neurological deficits or LAST, were observed during the 48-hour follow-up.

## Discussion

This prospective case series demonstrates that ultrasound-guided SeTB with SCN blockade can provide reliable surgical anesthesia for awake shoulder arthroscopy while preserving diaphragmatic function. The hybrid technique achieved a high surgical success rate with excellent intraoperative conditions, prolonged postoperative analgesia, and minimal reduction in DE. Although a 100% success rate was observed in this cohort, this finding should be interpreted with caution, given the limited sample size and study design.

Regional anesthesia techniques for shoulder surgery have evolved substantially over the past decade, with increasing emphasis on minimizing respiratory complications associated with the conventional ISB. Although ISB remains the most widely used regional anesthetic technique for shoulder procedures, its close anatomical proximity to the phrenic nerve frequently results in HDP. Previous investigations have demonstrated that phrenic nerve blockade occurs in a large proportion of patients receiving ISB, often leading to significant reductions in DE and pulmonary function [6,7,15]. These respiratory effects may limit the use of ISB in ambulatory surgery settings and in patients with reduced pulmonary reserve.

To address this limitation, several phrenic-sparing approaches to brachial plexus blockade have been proposed. Among these, the ST block has gained attention as a potential alternative technique for shoulder anesthesia. A systematic review and meta-analysis by Amaral et al. demonstrated that ST block provides effective analgesia for shoulder arthroscopy while significantly reducing the incidence of HDP compared with ISB [3]. Similarly, randomized trials and comparative clinical studies have reported that ST block can achieve analgesic outcomes comparable to ISB while preserving diaphragmatic function in a greater proportion of patients [16-18]. These findings support the concept that targeting the brachial plexus at a more distal level may reduce the risk of phrenic nerve involvement while maintaining adequate anesthesia for shoulder procedures.

SeTB represents a further refinement of distal brachial plexus blockade. By targeting the brachial plexus at the trunk level approximately 2-3 cm distal to the nerve roots, the injection site is positioned farther from the phrenic nerve, thereby potentially reducing the likelihood of diaphragmatic dysfunction. Karmakar et al. first described ultrasound-guided SeTB as a method capable of producing surgical anesthesia of the upper extremity while limiting phrenic nerve involvement [5].

Subsequent investigations have further explored the feasibility and anesthetic characteristics of this approach. Sivakumar et al. demonstrated the minimum effective anesthetic volume required for SeTB and reported reliable surgical anesthesia with favorable safety profiles [9]. Additional studies have confirmed that trunk-level approaches can provide effective regional anesthesia for upper extremity procedures while preserving diaphragmatic function [8,13]. The findings of the present study are consistent with these reports, demonstrating minimal reduction in DE and no clinically significant HDP following SeTB.

Another important consideration in awake shoulder arthroscopy is the occurrence of traction-related “cape pain,” which arises from dermatomes supplied by the SCN of the superficial cervical plexus. Conventional brachial plexus blocks may not reliably anesthetize these cutaneous branches, resulting in residual discomfort during shoulder traction. Anatomical and clinical studies have demonstrated that the SCN provides sensory innervation to the superior shoulder region and may therefore contribute to incomplete analgesia when only brachial plexus blockade is used [10-12]. The addition of a targeted SCN block may therefore enhance dermatomal coverage of the shoulder girdle and improve intraoperative comfort.

The hybrid SeTB-SCN technique used in the present study was designed to address this limitation. By combining trunk-level brachial plexus blockade with cervical plexus supplementation, the technique provides more comprehensive regional anesthesia of the shoulder region. In our cohort, all patients achieved adequate surgical anesthesia without conversion to general anesthesia, and intraoperative opioid requirements remained minimal. These findings suggest that the combined approach can effectively address both deep articular pain mediated by the brachial plexus and superficial traction-related pain originating from the cervical plexus. However, as a prospective case series without a control group or randomization, these findings demonstrate feasibility and clinical association rather than comparative efficacy or superiority.

Preservation of diaphragmatic function is particularly relevant in the context of ambulatory shoulder surgery. Enhanced recovery protocols emphasize early mobilization, reduced opioid consumption, and same-day discharge. Phrenic-sparing regional anesthesia techniques may therefore improve perioperative safety while maintaining effective analgesia. While the observed favorable diaphragmatic profile suggests potential utility in populations such as athletes or patients with pulmonary compromise, these groups were not directly studied in this cohort (ASA I-II only), and such applicability should be considered hypothesis-generating rather than conclusive.

In addition to athletes and physically active individuals, this approach may also benefit elderly patients or those with underlying pulmonary disease who may not tolerate diaphragmatic dysfunction induced by conventional interscalene block [19].

Despite these encouraging findings, several limitations should be acknowledged. First, as a single-center prospective case series without a control group, the study design limits direct comparison with other established regional anesthesia techniques and inherently carries the constraints of level IV evidence. Second, the relatively small sample size reduces the ability to detect infrequent complications associated with regional anesthesia. Third, DE was assessed using ultrasonography rather than formal pulmonary function testing. However, ultrasound is a widely accepted and practical surrogate for evaluating diaphragmatic function in regional anesthesia studies [7,12]; it may not provide the comprehensive functional assessment achieved with spirometry-based measures. Additionally, DE was not evaluated at 30 minutes post-block, and early hemidiaphragmatic paresis was not assessed, potentially limiting the detection of transient or early diaphragmatic changes. Collectively, these factors should be considered when interpreting the findings.

Future research should focus on randomized controlled trials comparing the SeTB-SCN technique with the interscalene block and ST block in patients undergoing shoulder arthroscopy. Such studies would help clarify the relative efficacy, safety profile, and optimal anesthetic volumes required for these techniques. Larger, well-designed randomized controlled trials are essential to validate these findings, assess reproducibility, and establish generalizability.

## Conclusions

This prospective case series demonstrates that ultrasound-guided SeTB, combined with SCN blockade, provides reliable anesthesia for awake shoulder arthroscopy, achieving 100% surgical success with stable intraoperative conditions. The technique resulted in effective analgesia, low opioid requirement, and prolonged postoperative pain control, supporting its utility in ambulatory practice. Importantly, diaphragmatic function was preserved, with no clinically significant HDP, highlighting its phrenic-sparing advantage. While these findings suggest that the SeTB-SCN approach is a viable alternative to the interscalene block, larger randomized controlled trials are needed to confirm comparative efficacy and safety.

## Appendices

**Table 1:** Baseline patient characteristic Data are presented as mean ± SD or n (%). SD: standard deviation, BMI: body mass index, ASA: American Society of Anesthesiologists

Parameter	Value
Number of patients	28
Age (years)	44.8 ± 17.5
Sex (male/female)	18/10
BMI (kg/m²)	26.4 ± 4.2
ASA I/II	16/12

**Table 2:** Perioperative outcomes following SeTB-SCN block Data are presented as n (%) or mean ± SD. SD: standard deviation, GA: general anesthesia, VAS: visual analog scale, SeTB: selective trunk block, SCN: supraclavicular nerve, DE: diaphragmatic excursion, HDP: hemidiaphragmatic paralysis

Parameter	Outcome
Surgical success (no GA conversion)	28 (100%)
Block onset time (minutes)	15.9 ± 3.0
Duration of surgery (hours)	1.88 ± 0.58
Intraoperative fentanyl (µg)	56.1 ± 12.4
DE reduction (%)	20.8 ± 1.7
HDP	0
Time to first rescue analgesia (hours)	17.0 ± 2.9
VAS score at 6 hours	0.2 ± 0.6
VAS score at 12 hours	1.2 ± 1.7
VAS score at 24 hours	4.0 ± 1.4
